# 1-(3-Phenyl­prop­yl)urea

**DOI:** 10.1107/S1600536809038501

**Published:** 2009-09-26

**Authors:** Yang Li, Guoxiong Hua, Alexandra M. Z. Slawin, J. Derek Woollins

**Affiliations:** a School of Chemistry, University of St Andrews, Fife KY16 9ST, Scotland

## Abstract

In the crystal of the title compound, C_10_H_14_N_2_O, double supra­molecular layers of PhCH_2_CH_2_CH_2_NHC(O)NH_2_ are formed parallel to the *bc* plane by inter­molecular N—H⋯O hydrogen bonding, with *R*_2_^2^(8) and *R*_2_^1^(6) motifs in the *b-* and *c*-axis directions, respectively. The mean plane of the C_ar_—C—C group makes a dihedral angle of 84.8 (2)° with the benzene ring.

## Related literature

For related structural information see Bernstein *et al.* (1995 ▶). For background chemistry, see: Gray *et al.* (2005 ▶); Hua & Woollins (2009 ▶); Renodon-Cornière *et al.* (2002 ▶).
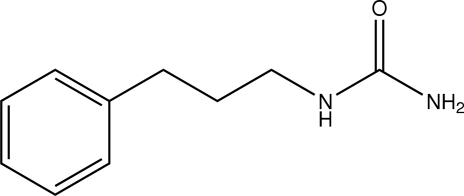


## Experimental

### 

#### Crystal data

C_10_H_14_N_2_O
*M* *_r_* = 178.23
Monoclinic, 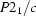

*a* = 17.002 (4) Å
*b* = 6.4953 (15) Å
*c* = 9.171 (2) Å
β = 91.401 (8)°
*V* = 1012.5 (4) Å^3^
*Z* = 4
Mo *K*α radiation
μ = 0.08 mm^−1^
*T* = 93 K
0.25 × 0.04 × 0.03 mm

#### Data collection

Rigaku Mercury CCD diffractometer
Absorption correction: multi-scan (*CrystalClear*; Rigaku, 2004 ▶) *T*_min_ = 0.981, *T*_max_ = 0.998
6696 measured reflections
2126 independent reflections
1360 reflections with *I* > 2σ(*I*)
*R*_int_ = 0.038

#### Refinement

*R*[*F* ^2^ > 2σ(*F* ^2^)] = 0.056
*wR*(*F* ^2^) = 0.144
*S* = 1.03
2126 reflections
122 parameters
H atoms treated by a mixture of independent and constrained refinement
Δρ_max_ = 0.23 e Å^−3^
Δρ_min_ = −0.20 e Å^−3^

### 

Data collection: *CrystalClear* (Rigaku, 2004 ▶); cell refinement: *CrystalClear*; data reduction: *CrystalClear*; program(s) used to solve structure: *SHELXS97* (Sheldrick, 2008 ▶); program(s) used to refine structure: *SHELXL97* (Sheldrick, 2008 ▶); molecular graphics: *PLATON* (Spek, 2009 ▶); software used to prepare material for publication: *SHELXTL* (Sheldrick, 2008 ▶).

## Supplementary Material

Crystal structure: contains datablocks I, global. DOI: 10.1107/S1600536809038501/bt5070sup1.cif

Structure factors: contains datablocks I. DOI: 10.1107/S1600536809038501/bt5070Isup2.hkl

Additional supplementary materials:  crystallographic information; 3D view; checkCIF report

## Figures and Tables

**Table 1:** Hydrogen-bond geometry (Å, °)

*D*—H⋯*A*	*D*—H	H⋯*A*	*D*⋯*A*	*D*—H⋯*A*
N1—H1*A*⋯O1^i^	0.88	2.10	2.936 (2)	159
N1—H1*B*⋯O1^ii^	0.88	2.09	2.8788 (19)	148
N2—H2⋯O1^ii^	0.863 (18)	2.127 (19)	2.9240 (19)	153.2 (17)

Symmetry codes: (i) 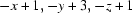; (ii) 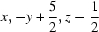.

**Fig. 1.**
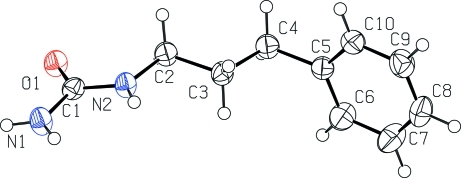
The molecular structure of the title compound with displacement ellipsoids drawn at the 50% probability level.

**Fig. 2.**
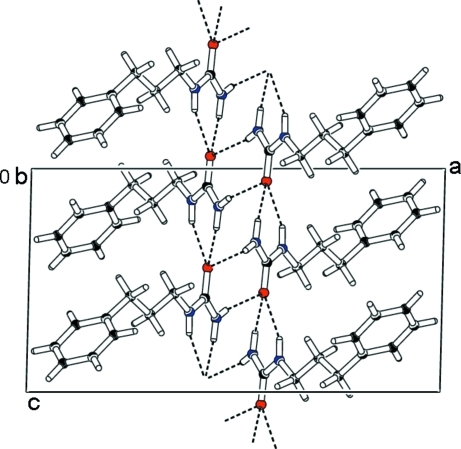
View along the b direction of the crystal packing of the title compound with hydrogen bonding shown as dashed lines.

## supplementary crystallographic information

### Comment

2,4-Bis(phenyl)-1,3-diselenadiphosphetane-2,4-diselenide [PhP(Se)(*µ*-Se)]_2_, which is known as Woollins reagent, WR, has received increasing attention due to its relatively unpleasant chemical properties and since it can be prepared readily and safely handled (Gray *et al.* 2005). Now it is becoming a very useful selenium source or reagent in synthetic chemistry (Hua *et al.* 2009; Hua & Woollins, 2009). We report here the synthesis and X-ray structure of C_6_H_4_(CH_2_)_3_NHCONH_2_. The title compound was prepared by the reaction of Woollins' reagent with 3-phenylpropan-1-amine.

Double supramolecular layers of the title compound (Fig. 2), PhCH_2_CH_2_CH_2_NHC(O)NH_2_, are formed parallel to the *bc* plane by intermolecular N—H···O hydrogen bonding (see Table 1), with *R*_2_^2^(8) and *R*_2_^1^(6) motifs in approximately b and c directions respectively (Bernstein *et al.*, 1995). The mean plane of the C3—C5 group makes dihedral angle of 84.8 (2)° with the benzene ring.

### Experimental

A toluene suspension of Woollins reagent (0.54 g, 1.0 mmol) and Ph(CH_2_)_3_NHCN (0.29 g, 2.0 mmol), which were prepared from cyanogen bromide with primary or secondary amine in dry methanol in the presence of excess of anhydrous CH_3_COONa at room temperature in almost quantitative yield (Renodon-Cornière *et al.* 2002), was refluxed for 4 h under nitrogen during which time the mixtures became a clear reddish brown solution. After cooling and addition of water (1 cm^3^) the reflux was continued for another 1 h. The solvent was removed in vacuum, and the residue was purified by column chromatography (silica gel, 9:1 dichloromethane/ethyl acetate as eluant) to afford the title compound in the yield of 95%. The single crystals of the title compound for the X-ray crystallographic analysis were obtained by recrystallization from dichloromethane/hexane as colorless blocks.

### Refinement

All H atoms except H2 (which was freely refined) were included in calculated positions (C—H distances are 0.98 Å methyl H atoms, 0.99 Å for methylene H atoms and 0.95 Å for aryl H atoms, N—H 0.88 Å) and were refined as riding atoms with *U*_iso_(H) = 1.2 *U*_eq_ (parent atom, amino, methylene and aryl H atoms) or *U*_iso_(H) = 1.5 *U*_eq_ (parent atom, methyl H atoms).

### Crystal data

**Table:**

C_10_H_14_N_2_O	*F*(000) = 384
*M**_r_* = 178.23	*D*_x_ = 1.169 Mg m^−^^3^
Monoclinic, *P*2_1_/*c*	Mo *K*α radiation, λ = 0.71073 Å
*a* = 17.002 (4) Å	Cell parameters from 2697 reflections
*b* = 6.4953 (15) Å	θ = 2.4–28.1°
*c* = 9.171 (2) Å	µ = 0.08 mm^−^^1^
β = 91.401 (8)°	*T* = 93 K
*V* = 1012.5 (4) Å^3^	Platelet, colorless
*Z* = 4	0.25 × 0.04 × 0.03 mm

### Data collection

**Table:**

Rigaku Mercury CCD diffractometer	2126 independent reflections
Radiation source: rotating anode	1360 reflections with *I* > 2σ(*I*)
confocal	*R*_int_ = 0.038
Detector resolution: 0.83 pixels mm^-1^	θ_max_ = 28.5°, θ_min_ = 3.4°
ω scans	*h* = −16→20
Absorption correction: multi-scan (*CrystalClear*; Rigaku, 2004)	*k* = −8→7
*T*_min_ = 0.981, *T*_max_ = 0.998	*l* = −9→11
6696 measured reflections	

### Refinement

**Table:**

Refinement on *F*^2^	Secondary atom site location: difference Fourier map
Least-squares matrix: full	Hydrogen site location: inferred from neighbouring sites
*R*[*F*^2^ > 2σ(*F*^2^)] = 0.056	H atoms treated by a mixture of independent and constrained refinement
*wR*(*F*^2^) = 0.144	*w* = 1/[σ^2^(*F*_o_^2^) + (0.0607*P*)^2^] where *P* = (*F*_o_^2^ + 2*F*_c_^2^)/3
*S* = 1.03	(Δ/σ)_max_ < 0.001
2126 reflections	Δρ_max_ = 0.23 e Å^−^^3^
122 parameters	Δρ_min_ = −0.20 e Å^−^^3^
0 restraints	Extinction correction: *SHELXL97* (Sheldrick, 2008), Fc^*^=kFc[1+0.001xFc^2^λ^3^/sin(2θ)]^-1/4^
Primary atom site location: structure-invariant direct methods	Extinction coefficient: 0.030 (5)

### Special details

**Table:**

Geometry. All e.s.d.'s (except the e.s.d. in the dihedral angle between two l.s. planes) are estimated using the full covariance matrix. The cell e.s.d.'s are taken into account individually in the estimation of e.s.d.'s in distances, angles and torsion angles; correlations between e.s.d.'s in cell parameters are only used when they are defined by crystal symmetry. An approximate (isotropic) treatment of cell e.s.d.'s is used for estimating e.s.d.'s involving l.s. planes.
Refinement. Refinement of *F*^2^ against ALL reflections. The weighted *R*-factor *wR* and goodness of fit *S* are based on *F*^2^, conventional *R*-factors *R* are based on *F*, with *F* set to zero for negative *F*^2^. The threshold expression of *F*^2^ > σ(*F*^2^) is used only for calculating *R*-factors(gt) *etc*. and is not relevant to the choice of reflections for refinement. *R*-factors based on *F*^2^ are statistically about twice as large as those based on *F*, and *R*- factors based on ALL data will be even larger.

### Fractional atomic coordinates and isotropic or equivalent isotropic displacement parameters (Å^2^)

**Table:**

	*x*	*y*	*z*	*U*_iso_*/*U*_eq_	
O1	0.56839 (7)	1.2854 (2)	0.55596 (11)	0.0367 (4)	
N2	0.61033 (9)	1.1114 (2)	0.35832 (16)	0.0321 (4)	
C5	0.81085 (11)	0.5672 (3)	0.32854 (16)	0.0325 (5)	
N1	0.54532 (10)	1.4186 (3)	0.33030 (14)	0.0401 (5)	
H1A	0.5219	1.5274	0.3665	0.048*	
H1B	0.5499	1.4060	0.2353	0.048*	
C2	0.64786 (11)	0.9469 (3)	0.44128 (17)	0.0342 (5)	
H2A	0.6075	0.8490	0.4749	0.041*	
H2B	0.6756	1.0048	0.5282	0.041*	
C10	0.79059 (12)	0.3888 (3)	0.25485 (18)	0.0396 (5)	
H10	0.7413	0.3258	0.2721	0.047*	
C6	0.88327 (12)	0.6552 (3)	0.3003 (2)	0.0451 (6)	
H6	0.8987	0.7777	0.3497	0.054*	
C3	0.70581 (11)	0.8358 (3)	0.34725 (18)	0.0338 (5)	
H3A	0.7425	0.9378	0.3063	0.041*	
H3B	0.6768	0.7703	0.2646	0.041*	
C4	0.75349 (11)	0.6713 (3)	0.42853 (18)	0.0354 (5)	
H4A	0.7828	0.7353	0.5114	0.042*	
H4B	0.7174	0.5671	0.4684	0.042*	
C9	0.84088 (13)	0.3000 (3)	0.1561 (2)	0.0457 (6)	
H9	0.8259	0.1765	0.1074	0.055*	
C8	0.91183 (13)	0.3890 (4)	0.1284 (2)	0.0475 (6)	
H8	0.9459	0.3288	0.0598	0.057*	
C7	0.93348 (13)	0.5670 (4)	0.2009 (2)	0.0533 (6)	
H7	0.9828	0.6294	0.1828	0.064*	
C1	0.57407 (10)	1.2717 (3)	0.42002 (17)	0.0305 (5)	
H2	0.6095 (11)	1.110 (3)	0.264 (2)	0.037*	

### Atomic displacement parameters (Å^2^)

**Table:**

	*U*^11^	*U*^22^	*U*^33^	*U*^12^	*U*^13^	*U*^23^
O1	0.0480 (9)	0.0409 (8)	0.0211 (7)	0.0078 (6)	0.0029 (5)	−0.0016 (5)
N2	0.0408 (10)	0.0356 (9)	0.0199 (8)	0.0082 (7)	0.0011 (6)	−0.0016 (6)
C5	0.0355 (11)	0.0340 (11)	0.0280 (9)	0.0034 (8)	0.0013 (7)	0.0026 (7)
N1	0.0575 (12)	0.0397 (10)	0.0232 (8)	0.0145 (8)	0.0041 (7)	−0.0004 (6)
C2	0.0405 (12)	0.0357 (11)	0.0264 (9)	0.0050 (8)	0.0023 (7)	0.0009 (7)
C10	0.0426 (13)	0.0348 (11)	0.0415 (11)	−0.0006 (9)	0.0055 (8)	−0.0006 (8)
C6	0.0402 (13)	0.0472 (13)	0.0480 (12)	−0.0050 (10)	0.0030 (9)	−0.0113 (9)
C3	0.0385 (12)	0.0351 (11)	0.0281 (10)	0.0043 (8)	0.0043 (7)	−0.0005 (7)
C4	0.0397 (12)	0.0363 (11)	0.0303 (10)	0.0037 (8)	0.0008 (8)	0.0013 (7)
C9	0.0541 (14)	0.0392 (12)	0.0440 (12)	0.0029 (10)	0.0029 (9)	−0.0079 (9)
C8	0.0447 (14)	0.0572 (15)	0.0408 (12)	0.0130 (11)	0.0071 (9)	−0.0058 (10)
C7	0.0388 (13)	0.0659 (16)	0.0557 (13)	−0.0033 (11)	0.0092 (10)	−0.0079 (11)
C1	0.0327 (11)	0.0361 (11)	0.0228 (10)	0.0010 (8)	0.0022 (7)	0.0001 (7)

### Geometric parameters (Å, °)

**Table:**

O1—C1	1.2559 (18)	C10—H10	0.9500
N2—C1	1.342 (2)	C6—C7	1.387 (3)
N2—C2	1.450 (2)	C6—H6	0.9500
N2—H2	0.863 (18)	C3—C4	1.525 (2)
C5—C10	1.380 (3)	C3—H3A	0.9900
C5—C6	1.388 (3)	C3—H3B	0.9900
C5—C4	1.514 (3)	C4—H4A	0.9900
N1—C1	1.344 (2)	C4—H4B	0.9900
N1—H1A	0.8800	C9—C8	1.367 (3)
N1—H1B	0.8800	C9—H9	0.9500
C2—C3	1.509 (2)	C8—C7	1.379 (3)
C2—H2A	0.9900	C8—H8	0.9500
C2—H2B	0.9900	C7—H7	0.9500
C10—C9	1.386 (3)		
			
C1—N2—C2	123.43 (14)	C4—C3—H3A	108.8
C1—N2—H2	115.5 (12)	C2—C3—H3B	108.8
C2—N2—H2	121.1 (12)	C4—C3—H3B	108.8
C10—C5—C6	117.78 (17)	H3A—C3—H3B	107.7
C10—C5—C4	120.97 (17)	C5—C4—C3	111.07 (14)
C6—C5—C4	121.11 (17)	C5—C4—H4A	109.4
C1—N1—H1A	120.0	C3—C4—H4A	109.4
C1—N1—H1B	120.0	C5—C4—H4B	109.4
H1A—N1—H1B	120.0	C3—C4—H4B	109.4
N2—C2—C3	109.73 (13)	H4A—C4—H4B	108.0
N2—C2—H2A	109.7	C8—C9—C10	120.42 (19)
C3—C2—H2A	109.7	C8—C9—H9	119.8
N2—C2—H2B	109.7	C10—C9—H9	119.8
C3—C2—H2B	109.7	C9—C8—C7	119.43 (18)
H2A—C2—H2B	108.2	C9—C8—H8	120.3
C5—C10—C9	121.23 (19)	C7—C8—H8	120.3
C5—C10—H10	119.4	C8—C7—C6	120.1 (2)
C9—C10—H10	119.4	C8—C7—H7	120.0
C7—C6—C5	121.1 (2)	C6—C7—H7	120.0
C7—C6—H6	119.5	O1—C1—N2	121.38 (15)
C5—C6—H6	119.5	O1—C1—N1	121.44 (16)
C2—C3—C4	113.74 (14)	N2—C1—N1	117.17 (14)
C2—C3—H3A	108.8		
			
C1—N2—C2—C3	−160.22 (17)	C2—C3—C4—C5	−179.52 (16)
C6—C5—C10—C9	0.2 (3)	C5—C10—C9—C8	−0.6 (3)
C4—C5—C10—C9	175.84 (17)	C10—C9—C8—C7	0.8 (3)
C10—C5—C6—C7	0.2 (3)	C9—C8—C7—C6	−0.4 (3)
C4—C5—C6—C7	−175.50 (18)	C5—C6—C7—C8	0.0 (3)
N2—C2—C3—C4	175.12 (15)	C2—N2—C1—O1	−2.2 (3)
C10—C5—C4—C3	−92.9 (2)	C2—N2—C1—N1	176.63 (16)
C6—C5—C4—C3	82.7 (2)		

### Hydrogen-bond geometry (Å, °)

**Table:**

*D*—H···*A*	*D*—H	H···*A*	*D*···*A*	*D*—H···*A*
N1—H1A···O1^i^	0.88	2.10	2.936 (2)	159
N1—H1B···O1^ii^	0.88	2.09	2.8788 (19)	148
N2—H2···O1^ii^	0.863 (18)	2.127 (19)	2.9240 (19)	153.2 (17)

Symmetry codes: (i) −*x*+1, −*y*+3, −*z*+1; (ii) *x*, −*y*+5/2, *z*−1/2.

## References

[bb2] Bernstein, J., Davis, R. E., Shimoni, L. & Chang, N.-L. (1995). *Angew. Chem. Int. Ed. Engl.* **34**, 1555–1573.

[bb3] Gray, I. P., Bhattachcharyya, P., Slawin, A. M. Z. & Woollins, J. D. (2005). *Chem. Eur. J* **11**, 6221–6227. 10.1002/chem.200500291 16075451

[bb4] Hua, G. & Woollins, J. D. (2009). *Angew. Chem. Int. Ed* **48**, 1368–1377. 10.1002/anie.200800572 19053094

[bb1] Renodon-Cornière, A., Dijols, S., Perollier, C., Lefevre-Groboillot, D., Boucher, J.-L., Attias, R., Sari, M.-A., Stuehr, D. & Mansuy, D. (2002). *J. Med. Chem* **45**, 944–954. 10.1021/jm011006h 11831907

[bb5] Rigaku (2004). *CrystalClear* Rigaku Corporation, Tokyo, Japan.

[bb6] Sheldrick, G. M. (2008). *Acta Cryst.* A**64**, 112–122. 10.1107/S0108767307043930 18156677

[bb7] Spek, A. L. (2009). *Acta Cryst.* D**65**, 148–155. 10.1107/S090744490804362X PMC2631630 19171970

